# “It's On Your Shoulders Now” Transitioning from Child-to-Adult UK Cleft Lip/Palate Services: An Exploration of Young Adults’ Narratives

**DOI:** 10.1177/10556656241236006

**Published:** 2024-03-04

**Authors:** Danielle McWilliams, Maia Thornton, Matthew Hotton, Marc C Swan, Nicola Marie Stock

**Affiliations:** 1 Centre for Appearance Research, 1981 University of the West of England, Bristol, UK; 2 Spires Cleft Centre, Level 2 Children's Hospital, 11269 John Radcliffe Hospital, Oxford, UK

**Keywords:** cleft lip, cleft lip and palate, cleft palate, counseling, familial adjustment, health policies, patient satisfaction, psychosocial adjustment, quality of life, transition of care, young adults

## Abstract

**Objectives:**

Treatment for cleft lip and/or palate (CL/P) in the United Kingdom is administered on a standardised pathway from diagnosis to early adulthood, with options to be re-referred in later life. At age 16, patients become responsible for their treatment decisions. Evidence from the wider health literature indicates this transition can be challenging and that this population may require additional support. The present study explored young people's experiences of transition to adult care in the context of CL/P services, with the aim of identifying support needs and informing future service delivery.

**Design:**

Individual semi-structured interviews were conducted with 15 individuals with CL/P (aged 17–25 years) to explore transition experiences. Interviews lasted an average of 69 min and data were analysed using reflexive thematic analysis.

**Results:**

Four themes, with subthemes, were identified: 1) Readiness for Transition covered feelings of preparedness and how health professionals approached transition; 2) Making Decisions as an Adult described concerns and considerations when making treatment decisions; 3) Finding and Using Support, reflected the roles of caregivers and peers in developing self-advocacy; and 4) Reflections on Transition Care offered insight into how care could be improved.

**Conclusion:**

Individuals born with CL/P may experience challenges in becoming responsible for their own care and treatment decisions. The findings of this study indicate that a dedicated transition protocol may be beneficial, such that adolescents are prepared to confidently access and manage their care into adulthood. Opportunities for improvements in transition planning and provision are discussed.

## Introduction

Treatment for cleft lip and/or palate (CL/P) was standardised in the United Kingdom (UK) following the 1998 Clinical Standards Advisory Group (CSAG) report, which found that care at the time was poorly organised and that treatment outcomes were consequently suboptimal.^1^ Every child born with CL/P in the UK is now automatically referred to one of 12 CL/P multidisciplinary networks and placed on a pathway of appointments, interventions, monitoring and surgery. The CL/P multidisciplinary team (MDT) typically includes clinical nurse specialists, surgeons, speech and language therapists, dentists, orthodontists, and psychologists.^2,3^ The National Health Service (NHS) Standard Contract for CL/P outlines a timeline which details when and how patients should receive care to ensure positive outcomes.^3^ CL/P treatment continues throughout childhood and often into early adulthood, with the option for adults to re-access the service in the future, often through a referral from a relevant health professional.

Individuals and families impacted by CL/P can also access support from charities such as the Cleft Lip and Palate Association (CLAPA), the leading UK charity. For young people navigating treatment, opportunities to meet with peer mentors, take part in residential weekends and join youth councils, in addition to online social and information events allow connection, support and discussion outside of a medical context.^4^ Peer support, defined as “*approaches through which people with similar long-term conditions or health experiences support each other to better understand the conditions and aid recovery or self-management*^5^*”,* has been found to create a sense of belonging and comfort for those in the CL/P community.^6,7^

Although prescribed treatment for CL/P may end in late adolescence,^3^ there is evidence of psychological and physical impacts of CL/P into adulthood. Adults with CL/P have reported feeling dissatisfied with their appearance^8,9^ and anxious about judgement by others.^10^ Previous studies have also identified potential difficulties in maintaining healthy relationships^6^ and physical health challenges.^11^ Concurrently, research has found that adults in the UK may be unaware of their entitlement to re-access CL/P treatment later in life, or may struggle to do so through their NHS General Practitioner (GP) or General Dental Practitioner (GDP).^11^

One way to ensure adults with CL/P feel they can re-access their CL/P team if they need it is by ensuring a comprehensive transition between child and adult services. When a child reaches 16 years old, they become responsible for their care and are required to formally consent to treatment.^12,13^ Until a child turns 16, a test known as the ‘Gillick test’ can be used by a clinician to ascertain whether a child understands a treatment, including its purpose, effects, risks and alternatives, enough to be able to consent to it without their caregivers’ input.^14^ Although a child who passes Gillick competency requirements can consent to treatment independently, those with parental responsibility can still override their refusal of treatment.^15^ The handing over of sole responsibility from caregiver to young person (age >16) means that they must sign consent forms, requiring them to be cognisant of the risks and likely outcomes of treatment, as well as their right to refuse.^13,16^ It is important that young people feel equipped to make their own medical decisions and have the information needed to do so when they reach 16 years old, such that they know who and where they can access support from if needed.

In 2016, the UK National Institute for Clinical Excellence (NICE) produced a set of recommendations to guide health professionals on how to prepare young people for this transition across all health teams.^17^ According to these generic guidelines, practitioners should begin planning for transition to adult care from when the child is 13 years old. The recommendations are divided into four sections: 1) transition services should be underpinned by overarching principles of patient involvement and choice, adopting a patient-centred and strengths-based approach to care, and involving general practitioners and regular reviews; 2) transition should be timed appropriately to the young person, with a named worker in place to support the process throughout and with an emphasis on developing decision-making independence; 3) transition should be supported by children's and adults’ services such that the young person knows who is in their care team and how to access them; and 4) young people should be followed up regularly by practitioners who are known to them to ensure they can access the support they would like. Within CL/P care specifically, transition to adult services is mentioned in the NHS Standard Contract for CL/P^3^, but without specific guidelines on how it should be implemented.

Although literature specifically addressing transition within CL/P care is limited within the UK population, young people in the United States of America have reported feeling “weighed down” by a “sudden responsibility” of decision-making and have struggled to seek support.^18^ It has also been indicated in UK- and Japan-based studies that perceived pressure to agree to treatment and a lack of discussion with healthcare professionals and parents/caregivers could be a barrier to this process.^19,20^

Young people with CL/P remain a relatively understudied group, particularly using data gathered examining young people's own perspectives.^21^ The present study explored young people's experiences of transition to adult care in the context of CL/P services, with the aim of identifying support needs and informing future service delivery.

## Method

### Ethical Considerations

Full ethical approval was granted by the University of the West of England Ethics Committee (Bristol, UK) before commencement of the study (approval reference HAS.20.12.063). British Psychological Society ethical guidelines were followed throughout.

### Design

Given the limited evidence base, an exploratory qualitative approach was taken. Individual, one-to-one interviews were carried out with young people born with CL/P in the UK. A semi-structured interview schedule (see supplementary table 1) was created, informed by NICE guidelines. A patient representative and two CL/P clinicians reviewed the schedule and their feedback was incorporated.

### Procedure

Participants were predominantly recruited via adverts on social media. Additionally, an information session was held for 16–18-year-old members of the CLAPA young people's council. Potential participants were initially invited to complete a brief online eligibility questionnaire. Those who met eligibility criteria (aged 16–25, born with and received treatment for CL/P in the UK) were sent an information sheet detailing the purpose and nature of the study, along with ethical information such as confidentiality and their right to withdraw. Interviews were facilitated by the second author, who is experienced in conducting qualitative research. Informed verbal consent was obtained before the start of each interview. Questions were open-ended, and prompts were used (eg, “can you tell me more about that?”) to elicit further information where answers were particularly brief, or the participant seemed uncertain on whether to elaborate. Interviews took place online via Microsoft Teams, using a combination of audio only (n = 3) and video calls (n = 12) dependent on participant choice. Interviews lasted an average of 69 min (range 44–96 min). Recordings of each interview were transcribed verbatim by an external transcriptionist.

### Data Analysis

Reflexive Thematic Analysis (TA) was used to analyse the transcripts. The first and second authors are research psychologists with training in qualitative methods. The senior author is a senior research psychologist with extensive experience in the field of craniofacial conditions. As per the six prescribed stages of TA, the authors became familiar with the data (1) and identified initial codes (2). Codes were then organised into themes and subthemes (3). Once the first and second authors agreed on themes, a discussion took place with the senior author (4), at which point, themes were finalised and named (5), and the final manuscript was produced (6).^22,23^

## Results

### Participants

Of the 20 people who expressed interest, 15 young adults (mean age: 21.4 years, SD: 2.44 years; 11 female) met the eligibility criteria and gave their consent to participate in the study. Participants were representative of eight of the possible twelve UK CL/P networks. Nine participants had been born with a cleft lip and palate, four had a cleft lip only, and two had a cleft palate only. Two reported having additional syndromes. Fourteen identified as ‘White British’ and one as ‘Mixed Race’. Eight participants were in either full- or part-time employment, five were studying at university and two were completing compulsory education.

### Presentation of Themes

**Figure 1.**
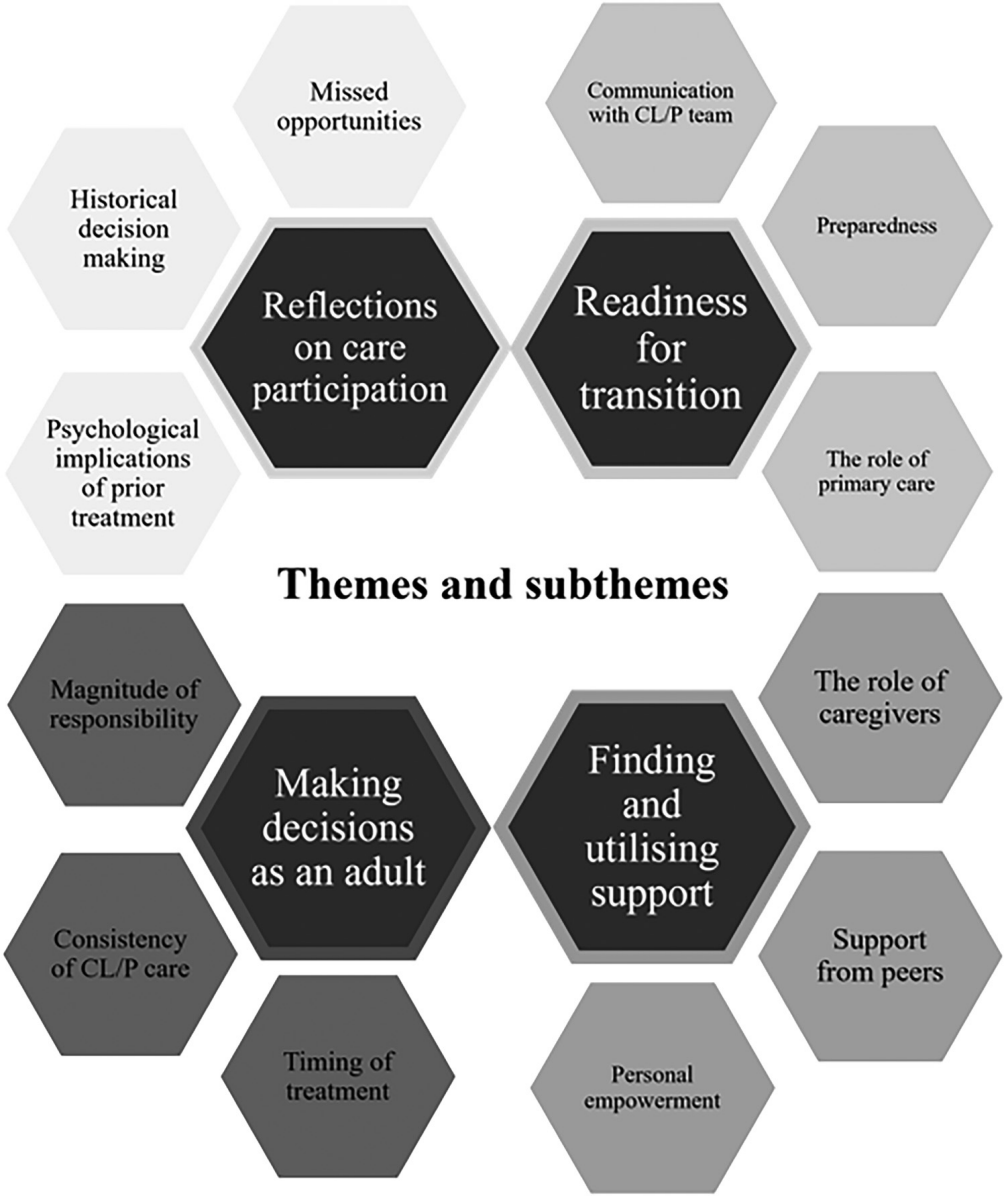
Diagram of themes and subthemes.

Four themes were identified, each with three sub-themes (see figure 1). Exemplar quotations are presented below, using ellipses to indicate shortened quotations and brackets to indicate added content to aid clarity. To maintain the anonymity of participants, pseudonyms are used, along with participant age and gender (M for male; F for female). Concurrent with TA guidelines,^22^ frequencies of themes and subthemes are not reported, but guidelines around quantifying language were adhered to^24^ such that “all” refers to all participants (or all but one), “most” indicates more than half and “some” indicates less than half but more than two.

## Theme 1: Readiness for Transition

The first theme discussed participants’ feelings of preparedness for transition to adult care and their experiences of how various health professionals approached the topic.

### Preparedness

When discussing how prepared participants had felt for the transition to adult care, some reported feeling familiar enough with the treatment process that they were able to begin making their own decisions: “*Because I’d…had quite a lot of surgery before [age 16], I think the knowledge did sink in and I was mentally prepared for [taking responsibility for treatment]”* (Luke, 22 M). For other participants, this was not the case and the shift in care had not been anticipated: “*There wasn’t really any gradual going up to it…and it was a bit daunting”* (Kirsty, 20F). Most participants reflected on the idea that time set aside to prepare them for making treatment decisions would have been valuable: “*If I’d just had a sit-down appointment with everyone who would be involved and got to know them…I would have felt a lot less out of my depth”* (Carrie, 24F). Some participants also felt that some MDT members were more sensitive to the challenges of transition than others: “*I spent the majority of my time with the speech therapist and they involved and spoke to me more than the surgeon”* (Reece, 22 M). Finally, all participants would have liked someone in the CL/P team to talk them through the differences between child and adult care: “*One nurse being available to me could have made a world of difference to my end of treatment”* (Anna, 19F).

### The Role of Primary Care

All participants reported a lack of confidence in their GPs and GDPs when it came to supporting them with decision-making during, and after, the transition period: “*I find GPs and dentists, they’re a bit out of the loop of everything”* (Will, 20 M). Most participants reported they had experienced problems with GDPs being unsure of how to treat them: “*My dentists never want to do anything to me because they’re scared to give me treatment”* (Ellie, 18F). Moreover, some participants had difficulties being referred to the CL/P team in early adulthood due to an apparent lack of GP understanding of their roles as referrers*: “I was trying to get a referral back to audiology and he said ‘Why?’ and I said ‘Well, I’ve got a cleft palate.’ He said ‘Well, why do you need a referral to audiology? What do you need that for?’ …[GPs] need to know this…it was a real battle”* (Louise, 22F).

### Communication with the CL/P Team

Experiences of interactions with specialist CL/P professionals regarding transition were mixed. Some participants reported being listened to: “*[My surgeon] was really nice…[they] didn’t make it seem like a confusing thing…[they asked what I wanted] and said “Oh yeah, I can achieve that for you”* (Rianna, 17F). One participant also reported that being given time to think about their treatment options was particularly helpful: “*I was given plenty of time to think about it, because they’d always come back to me in a few months, especially trying to decide on operations…I wasn’t pressured or rushed”* (Will, 20 M). In contrast, some participants felt hurried through decisions and that little consideration was given to their worries: “*When you’re younger they talk slowly and gently to you…[Now] they’re…straight to the point…No time to feel wobbly about it”* (Kirsty, 20F).

Some participants reported feeling intimidated during interactions with health professionals, and disempowered from taking part in decision-making: “*[Surgeon] was…intimidating…I kind of shut down a bit because I was like “they don’t understand, I don’t want to do this anymore”* (Rianna, 17F). One participant recounted a health professional expressing concern that their motivations for surgery were not sufficient: “*The surgeon said…‘Well, that's a big weight on my shoulders just to give you confidence…I’d prefer not to do it until you come back with a good reason’”* (Ellie, 18F).

## Theme 2: Making Decisions as an Adult

The second theme discussed participants’ feelings about the treatment decision-making process.

### Magnitude of Responsibility

In response to questions around treatment decision-making, participants reflected on the magnitude of responsibility they faced: “*When I was more in the driver's seat, I felt more nervous because…it's kind of on my shoulders now”* (Kirsty, 20F). Even when participants felt they had sufficient information, the decision whether to have treatment was significant: “*It's still hard to make a decision because they can be big choices”* (Will, 20 M). The shift in responsibility brought increased feelings of apprehension for some participants, due to the permanency of results, particularly with appearance-altering surgery: “*[as] someone that's lived with their same face looking back at you [in the mirror] for…19 and a half years, it's a difficult change”* (Georgia, 19F).

### Timing of Treatment

Some participants reflected that less consideration was given to the timing of treatment compared with childhood: “*I don’t think [my CL/P team] really understood that I like school and I like being with my friends…When you’re younger they respect it more often, but now they’re like ‘You can work around us. We don’t have a lot of time’”* (Rianna, 17F). For some participants, this had made being able to attend appointments difficult. Nonetheless, participants were aware that CL/P teams work under increasing time pressure and demand on resources: “*I guess [timing of appointments] was more a hospital resourcing problem than anything else and I guess if they’d had the time and resourcing, it might have been done differently”* (Carrie, 24F).

### Consistency in CL/P Care

Participants had varying levels of contact with their CL/P team throughout adolescence. For some, having consistency in their care was reassuring, as it allowed for trust to be built in an otherwise stressful environment: “*Even if you only see them for 20 min every few months… you end up getting to know them. More so because they’re fiddling around inside your mouth as well, you’ve got to get along with them”* (Luke, 22 M). In contrast, reports of participants receiving conflicting opinions from different team members had increased some participants’ anxiety: “*One [surgeon] does it this way, one person does it another way…I’m a bit worried about it”* (Oliver, 24 M). Finally, most participants offered some reflections on MDT appointments, and how they found it difficult to self-advocate: “*I hate [MDT appointments] because I just sit in a room and it's like a massive audience…I’m like ‘I don’t know who half of you are…I’m not going to just pop open my biggest secrets to you like, ‘yeah I hate myself, my nose is disgusting’”* (Ellie, 18F).

## Theme 3: Finding and Utilising Support

The third theme reflected on the role of peers and caregivers and explores the barriers and contributors to young people feeling empowered during transition.

### The Role of Caregivers

All participants viewed the involvement their caregivers had during the transition period favourably: “*Quite big things [e.g., at appointments; treatment, surgery decisions] could happen to me…and just having someone there to help me is good…[and] to have some advice”* (Rianna, 17F). The caregiver's role included advocacy, presence in appointments and offering their perspectives on medical decisions. Most participants reported their caregivers had been instrumental in providing guidance throughout the transition to adult care: “*It was my parents who really broke it down for me…If they weren’t there, I would have felt…like I don’t understand what's happening”* (Kirsty, 20F). Some participants, however, appreciated opportunities to speak with teams without their caregiver present: “*[Health professionals] would say ‘Would the parent mind stepping out for a little bit.’ I think it's a good thing to do that, because some people might be nervous to say certain things in front of their parents”* (Will, 20 M). Feeling ‘more adult’ without a caregiver present was reported, perhaps indicating a greater sense of autonomy: “*I think just having that one-to-one and feeling a bit more like an adult, it just made a big difference”* (Emma, 23F). Most participants expressed a preference for being addressed directly during communication with health professionals. Some participants gave the example of health professionals continuing to address the caregivers first, even when they had been managing their own care for years: “*Whenever we get letters and stuff it still says, ‘To the parent or guardian of …’ and I would be like ‘Why?! It's for me’”* (Jess, 24F).

### Support from Peers

All participants discussed being engaged in some peer support. Most participants had accessed this type of support through groups and events run by CLAPA and reported positive experiences: “*I spoke to someone about the option of jaw surgery because they’d gone through it…not many people can relate to it”* (Thea, 18F). Talking to other people with shared experiences also increased knowledge and empowered one participant to seek help for symptoms they would not have otherwise mentioned: “*At my next appointment I said, ‘this is a problem I’m having’. I never realised it was a problem until someone else mentioned it. So, that was the benefit of speaking to other people”* (Will, 20 M). Most participants also spoke about their passion for providing peer support to younger members of the CL/P community after experiencing it themselves: “*Not using my voice to help others navigate life with a cleft would be a disservice to myself and to younger kids going through it”* (Reece, 22 M).

### Personal Empowerment

Most participants shared ways that health professionals helped them to make informed decisions: “*They did have some little models [of faces before/after treatment] and that was always useful”* (Jess, 24F). There were also accounts which highlighted barriers to empowerment, with some participants feeling they had not been made aware of the available options. Most participants reported doing their own research online to try to find out more*: “At 16, I’d done my research on my own … on Google… It was quite depressing that's how I had to do it, but at least I knew”* (Ellie, 18F). One participant highlighted they had not been aware of a diagnosis they had received at birth, which they felt would have enabled them to understand their condition and symptoms better in adolescence: “*I found out once at an appointment, which was my 15-year review, that I was born with Pierre Robin Sequence as well, but I never knew that…It would have helped me make sense of everything”* (Thea, 18F)*.* Among all participants, expressions of having to work hard to advocate for themselves both in healthcare settings and in wider contexts were evident: “*I was born without a voice, and I created a voice for myself”* (Reece, 22 M).

## Theme 4: Reflections on Care Participation

The final theme offered insight into how early treatment experiences contribute to participants’ views about making treatment decisions later.

### Historical Decision-Making

Treatment decision-making in childhood had been a largely passive experience for most participants, in which health professionals had informed them about the care they would receive, rather than involving them: *“I had consultations where they told me what they were going to do… It was more relaying information. It wasn’t a conversation”* (Emma, 23F). Participants shared thoughts specifically about appearance-altering surgery, as they had not felt involved in these decisions at the time: “*With surgery that could change your appearance, there should be some level of consent… I think it's a really big thing to think about at 12/13…you don’t have a voice… I think that's quite a big thing that should be changed”* (Ellie, 18F). Most participants expressed rarely, or never, being asked what support they would like during the transition process: “*This interview is the first time [anyone has asked me how I feel about making medical decisions]”* (Luke, 22 M).

### Psychological Impact of Prior Treatment

Participants’ narratives included reflection regarding previous treatment experiences and the feelings these experiences evoked. Most participants felt they had needed specific support for their mental health and wellbeing when they were younger: “*It's not your average teenage depression and anxiety, it's a specific issue that needs specialist care… I went through such a lot and never got [specialist psychological support]”* (Louise, 22F). The anxiety that participants attributed to early consultations where decisions were not explained to them was reported by most to impede on their ability to manage their anxiety as adults: “*I’ve learnt as I go… you never really know what's going to happen and it makes me so anxious”* (Jess, 24F). Some participants’ reports could be indicative of symptoms of medical traumatic stress, occurring due to difficult treatment experiences and having a lasting impact into adult life: “*[The orthodontist] held me down and was forcing adult mouthguards into my mouth. I was screaming… I had such a bad experience it gave me anxiety about healthcare workers”* (Ellie, 18F). In some cases, this ongoing fear created a barrier to seeking further treatment: “*I couldn’t hear properly…but I didn’t want to tell anyone, because I was scared I would get another surgery”* (Ellie, 18F).

### Missed Opportunities

All participants reflected on experiences they wished had been handled differently in their treatment pathway. For example, some participants could not recall having psychological support: “*I’ve heard a lot recently about psychologists in cleft teams… That was never something anyone ever mentioned to me, and I really wish they had… That would have been life changing I think”* (Louise, 22F). Participants also spoke about their final MDT meeting, during which they had been ‘discharged’ from routine treatment. Most participants stated that they did not know how to re-access the CL/P team in future if they wished: “*They came to discharge me and they sort of just left me with nothing. I think if they’d have given me a direction or a number to get back in touch should anything happen, I would have felt a lot more confident”* (Carrie, 24F). Some participants reported that although they may have been asked if they wanted more input from the CL/P team going forward, they did not know what they were eligible for, making it difficult to feel in control: “*They didn’t say ‘here's what you could have.’ I don’t know what I don’t know”* (Matilda, 25F).

## Discussion

This study explored young people's experiences of transition to adult care in the context of CL/P services, aiming to identify support needs and inform future service delivery. Overall, findings suggest the NICE guidelines on transition from child to adult care are only partially being met in UK CL/P services and that improvements could be made to help young people feel more involved in their care.^25^ Challenges with transition could be barriers to developing independence and autonomy over treatment decisions. This could have lasting implications for accessing and engaging with treatment as an adult. A summary of the findings and subsequent recommendations are provided below and in Table 1.

**Table 1.** Summary of Key Findings and Recommendations.

**Theme**	**Subtheme**	**Summary of findings**	**Recommendations**
**1 Readiness for Transition**	1.1 Preparedness	Participants reported not fully understanding that they were becoming responsible for their own treatment decisions. As a result, participants did not feel that they had the opportunity to discuss transition or gather information to help them with the process.	Discussions about transition to adult care could be initiated by health professionals throughout adolescence, to set expectations about future changes within care and to allow individuals and families to ask questions and gather information. CL/P teams could consider allocating a named professional to act as a liaison and coordinator throughout the transition process.
	1.2 The Role of Primary Care	Participants would appreciate a greater awareness among General Practitioners and General Dental Practitioners of the CL/P treatment pathway in the UK and their role in the referral process.	Development of training and resources for non-specialist professionals may assist young people in receiving support before and during their transition to adult services. Resources to support young adults to better navigate and manage their own primary care could be a valuable addition to existing care.
	1.3 Communication with the CL/P team	Most participants reported that their experience of discussing prospective surgery had been positive and they could recall times where they felt listened to. Some participants experienced feeling dismissed or rushed during conversations about their care.	Whilst it is important that parents and caregivers are informed about their child's care, wherever appropriate, facilitating conversations about care and treatment in a developmentally appropriate and accessible way for the young person may empower them to feel more part of the decision-making process.
**2 Making Decisions as an Adult**	2.1 Magnitude of Responsibility	Participants shared that they felt a great sense of responsibility when faced with deciding on care, which could be overwhelming.	Psychological services input should be an integrated part of medical decision-making, to help individuals to work through apprehensions and motivations.
	2.2 Timing of Treatment	Participants recognised the multiple factors involved, both for themselves and the CL/P teams, when deciding when is the right time for surgery. School, university, work and missing social plans were discussed as important considerations for timing of care.	Being aware of possible changing motivating factors behind treatment may be important for helping young adults make decisions about care. Maintaining an awareness that young adults have priorities that could be different to those taken into consideration for children may pre-empt and support discussions about timing of treatment.
	2.3 Consistency in CL/P care	For some participants, receiving conflicting advice from different members of their CL/P team added to the anxiety, and reduced confidence levels when considering decisions. Some participants cited MDT meetings as especially difficult, especially due to the unfamiliar clinicians in the room. This prevented some participants from feeling safe to speak openly.	Where possible, individuals should be familiar with the team they are meeting with in appointments and introductions to new/key treatment team members should be part of the transition process. MDT meetings could be preceded or followed by a conversation with a known and trusted CL/P team member to check that the individual is comfortable and if there is anything they would like to discuss individually. A named transition professional could facilitate introductions and act as a consistent figure throughout transition. Psychologists having capacity and resources to regularly review young people (eg, at audit points) could make them a more accessible and visible presence for young people to seek support if it is needed.
**3 Finding and utilising support**	3.1 Support from Peers	Most participants had some contact with peers through the Cleft Lip and Palate Association (CLAPA), which they found helpful and affirming. Most participants spoke about seeking opportunities to provide peer support to others.	The benefits of engaging in networks within CLAPA and social media could continue to be emphasised by CL/P teams alongside their own peer support initiatives where appropriate.
	3.2 Personal Empowerment	Most participants said that they had done a lot of their own online research as they did not feel that they had enough information to make decisions about their care alone. Some participants spoke about different ways that information was provided which helped them to feel informed, such as by looking at models and photos.	A combination of shared-decision making and information-sharing within treatment, clinical psychology input where indicated and encouragement of peer support engagement could contribute towards feelings of empowerment for those seeking or undergoing treatment.
	3.3 The Role of Caregivers	All participants felt that they had good support from their caregivers throughout the transition process. Some participants shared that they appreciated being given space to talk to health professionals without their caregivers present.	Caregivers, as the primary source of support for most young people, could face challenges with transition themselves. There may be a need for clinical psychology input for these caregivers. Young adults should be consulted regularly about their wishes regarding their caregivers’ involvement in their treatment, such as being present for appointments and arrangements whilst they are staying in hospital.
**4 Reflections on transition care**	4.1 Historical decision making	Most participants felt that receiving care had been a largely passive experience throughout their childhood and adolescents. Reflections on feeling unheard about appearance-altering surgery during childhood were shared as particularly difficult.	With the support of psychologists, it is important to continue to find ways to involve children in their treatment as much as is feasible, with the view to reducing the traumatic impact of treatment from a young age. Clinical psychology input throughout transition should be sensitive to the possibility that the increase in responsibility for treatment, may reveal or heighten post-traumatic symptoms resulting from childhood experiences.
	4.2 Psychological impact of prior treatment	Some participants shared experiences of childhood treatment which had been particularly traumatic and disempowering such as being ‘held down’ and/or not being fully informed about what was happening.	•
	4.3 Missed Opportunities	All participants reflected on aspects of their care which could have been more supportive throughout their transition from child to adult CL/P care. Most participants who had been discharged from their CL/P team spoke about this experience as confusing and disempowering as they did not know what further support they could be entitled to.	As a young adult approaches discharge, ensuring that there are multiple opportunities and forums for them to ask questions and gather information in a familiar environment could enable individuals to feel heard and able to share their concerns. It is important that young adults leave CL/P services with information about what is available to them and how to access it.

### Improving Young People's Understanding of ‘Transition’

Although the medical age of consent is 16 in the UK,^13,16^ the findings of the current study indicate that young people may not be aware they are able, and required, to make treatment decisions independently from this point. While some participants reported feeling ‘listened to’ as they approached adulthood and believed that health professionals had taken their treatment goals into account, most were not fully aware that a ‘transition’ had occurred and did not recall a member of their CL/P team explaining that anything had changed. Participants subsequently felt underprepared for taking responsibility for significant treatment decisions. In line with the NICE guidelines,^17^ these findings suggest that young people require an understanding of what transition to adult care is, when it will happen, why it happens, how it will be managed, and by whom.

### Facilitating Young People's Participation in Care

Most participants in the study indicated that experiences with previous treatment may have prevented them from accessing the treatment that they would like in the future. Some participants reported traumatic experiences during childhood that continued to impact on them. This is concurrent with existing literature citing that anxiety, often stemming from traumatic experiences with treatment and surgery, contribute to adults’ decisions not to have further surgery.^26–28^ Having to undergo surgery and treatment, irrespective of invasiveness/traumatic association, without being involved in decisions during childhood has also been shown before to cause a feeling of ‘treatment burnout’^27,29,30^ which was inferred by some participants, who spoke of a hesitancy to seek treatment that they feel they need. A recent review of long-term illness literature indicated that children as young as eight years old would have liked to be provided with more information about any proposed treatment to help them to understand and offer an input into treatment decisions.^31,32^ Children's accounts of this process led to conclusions that their ability to contribute to decisions were routinely underestimated.^32^

Shared decision-making is defined as ‘patients and professionals working together to create a mutually agreed treatment plan’.^33^ Across healthcare research and practice more generally, the notion of shared decision making has been demonstrated to be the most empowering way to enable patients to have their voices heard and to stay in control of their treatment.^34^ Shared decision making is a collaboration between patients and professionals, incorporating patients’ goals, expectations and priorities along with the most medically appropriate care.^33^ Broader health literature indicates that employing this approach with child and adolescent patients encourages patient autonomy and involvement and promotes self-confidence and self-efficacy in young people as they feel heard and respected.^19,35^ Within CL/P services, a continued effort towards shared-decision making approaches is recommended. Furthermore, using uncomplicated language and consistently addressing the patient, as opposed to their caregivers^17,20,36^ are ways that CL/P professionals can continue to support transition for caregivers and adolescents, which were not consistently implemented with the current sample.

When making decisions about CL/P surgery and treatment, several factors and potential motivations may be at play. Throughout childhood, most interventions are carried out with the aim to improving a function (eg, feeding, hearing or speech^2,3^). In adulthood, more treatment focuses on appearance.^3^ Some participants believed that appearance-altering surgery would improve their confidence, so it was important for them that the timing of surgery be considered in the context of their changing social world. Although surgery is not always the most appropriate course of action, it is important that these goals and motivations are taken into consideration by CL/P teams to acknowledge what is important to their patients, in line with NICE guidelines^17^ and recommendations for shared decision making.^19,33,37,38^ It is well documented that adolescence and early adulthood is a socially formative, yet often uncertain time, so motivations for appearance-altering surgery being rooted in social environment and feelings of wanting to improve self-confidence are unsurprising, and supported by other CL/P literature.^19,26,27,39^

Many of the concerns raised by participants in the current study related to communication with health professionals, feeling informed and being involved in treatment decisions. Participants suggested that having an allocated transition worker would have helped them to feel heard and involved. This is another of the key NICE recommendations^17^ and is supported by broader health literature, which suggests that a named worker improves overall patient experience, helps caregivers to feel more at ease, increases patient engagement and understanding of treatment, aids in decision making and improves access to appropriate care for adolescents and young adults.^40–44^ Having a clinical nurse specialist adopt the transition worker role has been shown to aid in the communication of medical information for young people undergoing extensive treatment for other conditions.^45^ Within CL/P care, clinical nurse specialists play a significant role in early care, often supporting families from pre-natal diagnosis until after primary surgeries are completed during infancy.^3^ The role of the clinical nurse specialist is highly valued, both by CL/P teams^46^ and amongst families,^47^ particularly for their role in providing a consistent and reassuring presence for families undergoing significant changes. For adolescent and adult CL/P care, a comparative figure within transition care could be extremely beneficial.

Another way to support patients in making treatment decisions is to consider the roles their peers and caregivers may have played in the past and may still be playing once the young person reaches age 16. In the current sample, it was mostly caregivers, rather than healthcare professionals, that equipped and supported participants to take ownership of their treatment. It is possible that the recruitment material for this study reached young adults who predominantly had a supportive network throughout their treatment such that they felt able and motivated to engage with support, but it must be recognised that not all adolescents have positive familial support. Pressure from caregivers has been reported in other CL/P literature as a concern for young people making treatment decisions,^19,20^ so an over-reliance on caregivers to support the transition process could carry risks of young adults being unsupported, consenting to, refusing or not being able to access treatment against their wishes. Caregivers also may underestimate their child's feelings of being ‘left out’ of CL/P treatment decisions,^48^ thus could benefit from support to understand how to facilitate their child's appropriate involvement in care. It is also indicated in wider health literature that caregivers may require support themselves with the stress and trauma associated with their child's ongoing care, especially given their responsibility for consenting to treatment.^49,50^ Caregivers may also benefit from support around relinquishing this responsibility, and with anxiety about their child's future^29,51^ and how to best support them.^52,53^

All participants in this study had engaged in peer support which were cited here and in existing research to decrease feelings of being ‘alone’ and provide assurance that others understand their experiences.^6,7^ CL/P teams continuing to emphasise and promote the opportunities provided by CLAPA and within their own teams where appropriate could increase engagement and uptake of peer support initiatives.

The NICE recommendations for transition from child to adult care in health services emphasise the importance of GPs in supporting transition.^17^ For most CL/P services, a referral from a GP or GDP is needed for an adult who has previously been discharged to return to care.^3^ It is important, therefore, that these health professionals are equipped with the knowledge and information they need to fulfil this role; referring and advising their patients accordingly. However, adults with CL/P have reported both in previous research and in the current study that their GPs and GDPs do not seem aware of the re-referral process and can be reluctant to offer them treatment and support with their care.^6,10,27^ If primary care practitioners were involved in a transition process for young adults, they may be better informed and equipped to support their patients after discharge from their CL/P team. It is customary for GPs to be sent copies of clinic letters and follow-up communications with patients after appointments, so if transition was routinely discussed in clinics, this information could be reflected in the letters, creating a cascade of information from CL/P team to young person and their GP. Participants in the current study could perhaps have benefitted from accessible resources aimed at young people and adults to provide to their GP and/or GDP regarding their role as referrers. In 2020, CLAPA created a referral letter template for adults with CL/P to provide to their GPs to use as part of the ‘Returning to Care’ pack which is now distributed to young people approaching transition,^54^ but for those who turned 16 prior to its development, there may remain a gap in knowledge about GP/GDPs roles and what support is available.

The finding from prior research that adults may not be aware of the option to re-access CL/P care indicates that opportunities to provide information were missed. Therefore, it may be useful and appreciated by patients for CL/P teams to attempt to re-engage with them as a reminder that they could still access support. It is recognised that individually contacting every discharged patient from each CL/P team would place huge administrative burden on teams and would likely still ‘miss’ people whose details are not up to date. However, adults could be reached via leaflets in GP or GDP waiting areas, posters or distributing social media posts through NHS Trusts or individual GP/GDP practices.

### Supporting the Transition Process

One way in which CL/P team can monitor and mitigate the impact of past and present treatment on young adults, along with other challenges around transition is through Clinical Psychology input. Clinical psychologists were introduced into CL/P MDTs in response to the growing body of literature on the impact of CL/P on emotional and psychosocial health.^55–58^ The present findings suggest that young adults did not feel that they had been spoken to about what it means to be an adult in the context of their care and that many had negative experiences of treatment in childhood but had not seen the clinical psychologist in their team. This indicates room for growth in the reach of psychologists already in CL/P teams, perhaps in terms of staffing,^59^ to enable more young people to access support to think about the treatment options available and the potential outcomes. One way to operationalise this could be to ensure adequate time and capacity for psychologists to meet patients and consult at each audit point (ie, at ages 5, 10 and 15) as standard, thus familiarising patients and families with the psychologist in the team. While commonplace in many UK cleft services, some services experience reduced clinical psychology capacity which can result in reduced opportunities for clinical psychologists to review young people as they grow through adolescence. Psychology reviews at these time points may empower young people to ask for and access support when they feel they would like it.

Whilst some participants in the current study had been in contact with a psychologist in the CL/P team, others could not recollect seeing one, and some were unaware that they could have sought this support. This lack of knowledge about adult provision is documented in previous, larger-scale CL/P literature,^27^ and has culminated in feelings of abandonment by CL/P teams.^18^ Patients can be discharged without an understanding of what is available to them, or even that this means they will not routinely return to clinic,^18,27^ which was also reflected in our findings. Some participants recalled their discharge meeting during their interviews, citing a room full of professionals that they did not know asking personal questions and talking ‘at’ them. Although it is possible that information about returning to care may have been provided at this point, it may not be the most appropriate environment compared with the gradual, collaborative transition process suggested by NICE. This concurs with existing literature that large MDT clinic appointments with multiple professionals in one room with the individual/family, whilst useful for treatment planning and collaboration, can also be uncomfortable for patients and make processing information and building rapport with professionals difficult.^60–62^ Providing space for a young person to meet separately with a known member of their MDT before or after their discharge meeting may help individuals to feel more at ease and able to share concerns, gather information and ask questions.

Transition resources to support young people with other health conditions do exist. For example, ‘Ready Steady Go!’^63^ is a series of booklets aimed at young people with ongoing physical health needs (eg, asthma) preparing to take control of their treatment. The goal is for young people to collate the information they need to feel confident in making treatment decisions with the support of their healthcare professionals, instead of relying on caregivers, which responds to the NICE guidelines for ensuring a smooth transition process for young people. Although this resource has been adapted to be specific to some conditions,^63^ the components of the booklets are not applicable to all long-term conditions. CL/P care is potentially lifelong in terms of healthcare provider input but comparatively low maintenance in terms of everyday management (compared with the medication regimes for asthma or diabetes, for example). Dedicated transition programmes for young people with heart disease^64^ have been shown to improve patient experience, reducing the amount of time young adults spent waiting for appointments and increased knowledge of how to describe and manage their condition.^65^ Existing transition resources across other services include information for both adolescents and caregivers about caregiver involvement, facilitating the potentially sensitive conversations around support from caregivers becoming more emotional as opposed to practical, decision-making responsibility.^52,63–65^

Some UK CL/P teams have downloadable transition resources on their websites.^66,67^ CLAPA have produced a ‘Returning to Care’ package with booklets, templates and leaflets about the support that adults are entitled to after discharge from their team.^54^ This package was evaluated and pilot recipients reported feeling more confident in re-accessing care and more likely to engage with CLAPA's information and support services.^68^ This pack is now being distributed to CL/P teams across the UK for use in clinics. Although this pack is not a transition protocol, it does provide important information to young people with CL/P preparing for adult care.

The importance of preparing young people for the transition to adult care is recognised in the NHS Standard Contract for CL/P care,^3^ as well as in international CL/P guidelines.^69^ Additionally, it is acknowledged that poorly managed transition can result in non-adherence to treatment and loss of contact with patients.^3^ However, no standardised guidance on transition management is provided. As a consequence, transition practices differ across cleft teams, which is out of line with the philosophy behind the centralisation of CL/P. Development of a manualised CL/P-specific transition protocol may prove difficult due to the nuances of individual teams, including the size and structure of each team, local policies, resources available and appearance of transition itself (for example, some CL/P service sites are “lifespan” and other services require patients to move from a children's hospital to an adult provision). Thus, whilst a UK-wide uniformed process may not be realistic, an adaptable template for CL/P professionals on how to manage transition with young people and families, including when this should be started and who should be involved, may be welcomed by the community and support the implementation of NICE guidelines.

## Methodological Considerations

This study offers insight into the experiences of young adults moving from child to adult CL/P care in the UK. Elaborating on existing transition literature in other health conditions, it provides information for health professionals on areas of strength of current services, in addition to what challenges their patients may be facing at this time, and the subsequent recommendations could be used to inform service development.

Most participants were white females, which limits the ability for cultural or gender considerations to be discussed in this paper. Additionally, those with a cleft palate only were underrepresented and no participants had additional learning needs, which could have prevented issues or considerations relating to disability being explored within transition care. The study also only heard the voices of patients with CL/P; it may be helpful for future work to consider the views of caregivers and CL/P professionals.

Another consideration that underpinned the data analysis and discussion, was that some of the terminology used commonly in healthcare and in the NICE recommendations was not familiar to the young people in the study. Most of the participants in the current study did not identify with the term ‘transition’ and did not feel that they had gone through this process, so the interviewer was mindful of this when asking follow-up questions. There is also significant heterogeneity in the amount of treatment individuals with CL/P will need, varying according to cleft type, orthodontic and speech outcomes and satisfaction with appearance. This means that some adolescents are likely to be more familiar with the CL/P team than others, who may only attend appointments for MDT reviews and audit, several years apart.^4^ Most participants were also recruited through a cleft charity or related social media and so may have different perspectives to those who are not involved or aware of third-sector support. The findings from this study were collated and analysed with these potential issues in mind.

## Conclusion

Transitioning from child to adult CL/P care can be a challenging time for young adults, attempting to navigate their own needs, goals and potentially challenging feelings whilst finding their place within a wider healthcare team. Although the present study demonstrates that peer and caregiver support has been helpful and that some CL/P professionals have been sensitive to the needs of patients at this time, experiences of a ‘transition period’ or process were lacking. This resulted in some young adults feeling unsure of where to turn for treatment or support. Unmet needs within this group and possible missed opportunities for shared decision making may indicate that some young people are misinformed about their ability to choose or refuse treatment, whilst others may not be accessing the care they are eligible for. As such, it is recommended that work towards unified guidance on transition be considered, taking lead from existing resources and literature, to ensure that those preparing for adulthood are well-equipped for adult care. We would also hope that future transition work included outreach to already-discharged adult patients, such that the offer of further treatment is accessible for all adults with born with a CL/P.

## Supplemental Material

sj-docx-1-cpc-10.1177_10556656241236006 - Supplemental material for “It's On Your Shoulders Now” Transitioning from Child-to-Adult UK Cleft Lip/Palate Services: An Exploration of Young Adults’ Narratives Supplemental material, sj-docx-1-cpc-10.1177_10556656241236006 for “It's On Your Shoulders Now” Transitioning from Child-to-Adult UK Cleft Lip/Palate Services: An Exploration of Young Adults’ Narratives by Danielle McWilliams, Maia Thornton, Matthew Hotton, Marc C Swan, and Nicola Marie Stock in The Cleft Palate Craniofacial Journal
