# Supplementary material for: “It's On Your Shoulders Now” Transitioning from Child-to-Adult UK Cleft Lip/Palate Services: An Exploration of Young Adults’ Narratives
Source: Cleft Palate Craniofac J. 2024 Mar 4;62(6):1039–52. doi: 10.1177/10556656241236006 (PMC12120205; doi:10.1177/10556656241236006)
Supplement: sj-docx-1-cpc-10.1177_10556656241236006 - Supplemental material for “It's On Your Shoulders Now” Transitioning from Child-to-Adult UK Cleft Lip/Palate Services: An Exploration of Young Adults’ Narratives [file sj-docx-1-cpc-10.1177_10556656241236006.docx]

**Table 1 –** Summary of semi-structured interview guide.

| Section | Question |
| --- | --- |
| Experiences of involvement in treatment | How much were you involved in your care while you were growing up? |
|  | What – if anything - do you feel the cleft team did to involve you in your care while you were growing up? |
|  | How much are you involved in your care now? |
|  | If applicable - what are the main differences between the involvement you had as a child and the involvement you have now? |
|  | What – if anything - do you feel the cleft team do to involve you in your care now? |
|  | Do you feel like you have enough information and support to manage your own cleft treatment moving forward? If you need any more information in the future do you know where to get it? Where would you go? |
| Timing and review of transition | How old were you when you felt like you became more involved in your cleft treatment? |
|  | Did you feel prepared to manage your own treatment at that time? If no, why not? If yes, what helped you to feel prepared? |
|  | Has anyone ever asked you how you feel about making these decisions? If yes, who, when, how? Was this reviewed at any point? |
|  | What has been your experience of other health professionals outside of the cleft team during this time in your life, for example your GP, your dentist? |
| Support with transition and decision making | Have you ever been allocated a named person (a nurse, for example) to be your port of call if you have any questions or concerns about moving from child to adult cleft care? |
|  | Would having a named person have been helpful for you? What would they have been useful for? |
|  | Did anyone else support you with your move to adult care? (e.g. GP) |
|  | Have you ever been offered support from CLAPA or another young adult with cleft to help you move to adult care? |
|  | Has anyone ever helped you to share your views at moments when you found it difficult to understand your care or found it difficult to speak up? |
|  | Have you been given the option of using mobile technology, apps or email to stay in contact with your cleft team? |
|  | When you move/moved from child cleft services to adult services, will you be/were you given a way to stay in touch with key people and/or networks you had growing up? |
|  | Has your cleft team/CLAPA mentioned any peer support opportunities to you (chances for you to speak with other young adults impacted by cleft about the transition process or treatment in general? |
| Independence in managing treatment | Have you ever been asked if you’re comfortable with your parents being involved in your treatment choices/coming to appointments with you? How do you feel about your parents’ involvement? |
|  | Have you been given the opportunity to ask questions or raise concerns without your parents being in the room with you? |
|  | Do you feel your wishes about your parents’ involvement has been listened to and respected? |
|  | If the child team is different from the adult team, did you meet anyone from the adult team before you moved? |
|  | Were you given any information about your past treatment, your medical records or your cleft ‘journey’ in preparation for you moving into adult cleft services? |
|  | Did anyone ever discuss with you what the main differences are between child cleft services and adult cleft services? (E.g. signing your own consent forms, making the decisions around treatment yourself, being ‘in control’ of what happens to you and when, being in a different hospital) |
| Review of care and experiences | If applicable, since you have ‘transitioned’ to adult care (turned 16), would you say your care has been consistent? Have you seen the same people each time? |
|  | If you’ve missed any appointments, have they followed up with you? |
|  | Do you know how to get back in touch with the cleft team if you needed to? |
|  | *If the participant has not engaged in any cleft services since they ‘took control’, ask* Has anyone been in touch with you from the cleft team to check that you do not want any further treatment, and to let you know how to contact them in the future? |
|  | Overall, do you feel like the move from child to adult cleft care has been successful for you? |
